# Brightness of AlGaInAs/InP Multimode Diode Lasers with Different Aperture Widths

**DOI:** 10.3390/nano13202746

**Published:** 2023-10-11

**Authors:** Yulia Kirichenko (Bobretsova), Dmitriy Veselov, Alexander Klimov, Sergey Slipchenko, Natalia Shuvalova, Andrey Lyutetsky, Nikita Pikhtin, Alexander Marmalyuk, Vladimir Svetogorov, Yuriy Ryaboshtan, Maksim Ladugin

**Affiliations:** 1 Ioffe Institute, 194021 Saint-Petersburg, Russia; 2 Sigm Plus Company, 117342 Moscow, Russia

**Keywords:** AlGaInAs/InP heterostructure, ultra-narrow waveguide, lateral brightness, optical power

## Abstract

A set of semiconductor lasers with different stripe widths is fabricated based on the AlGaInAs/InP heterostructure with an ultra-narrow waveguide. The key characteristics of the lasers (light-current curves (L-I), current-voltage curves (I-V), and spectral and spatial characteristics) are measured, and their dependence on the stripe width is shown. The operating optical power increases from 1.4 W to 4.3 W; however, the lateral brightness decreases from 1.09 W/(mm*mrad) to 0.65 W/(mm*mrad) as the stripe width increases from 20 to 150 μm.

## 1. Introduction

Semiconductor lasers operating at 1550 nm are widely used in laser ranging, LIDAR, fiber optic communication, solid state laser pumping and other systems. These lasers have a number of advantages over lasers emitting at other wavelengths. An emission near 1550 nm is less attenuated in the atmosphere and is also considered relatively eye-safe. Also, this emission is in the transparency window of fused silica, so it is possible to transmit data signals over long distances via fiber optic communication lines with minimal loss. In addition, an emission near 1550 nm is used in medicine and in various scientific applications such as spectroscopy and material analysis. The study and development of 1550 nm diode lasers offers many benefits and can lead to new advances in various industries.

For most applications, high-power diode lasers are of greatest interest. First of all, the laser power is determined by the heterostructure design. Recent studies have shown that lasers based on InGaAs/AlGaInAs/InP heterostructures with ultra-narrow waveguides have a higher optical power [1]. By using the ultra-narrow waveguide design, the heat sinking from the active region can be improved; internal optical loss, and series and thermal resistances can be reduced; and as a result it becomes possible to maintain the linearity of the L-I curve up to higher pump currents. Nevertheless, the diode laser maximum power is limited by internal physical phenomena and processes [2,3]. Therefore, a further power increase for lasers of this design is possible due to stripe contact (aperture) widening, which can be seen from Equation (1):

(1) $$P_{out}=\left(1-R_{L}\right)\upsilon_{g}W\;\hbar \omega \frac{d_{qw}}{\Gamma_{qw}}\;S_{f}(L)$$

where $R_{L}$ is a front mirror reflectivity (light is emitted through the front mirror, rear mirror reflectivity ~100%), $\upsilon_{g}$ is the group velocity, *W* is the stripe width, ℏω is the photon energy, $d_{qw}$ is the quantum well thickness, $\Gamma_{qw}$ is the active region optical confinement factor, $S_{f}(L)$ is the density of photons propagating in the forward direction along the cavity axis and *L* is the cavity length [4]. Based on Equation (1), the output optical power is directly proportional to the stripe width, which is clearly shown in [5].

In addition to high optical power, the spatial beam quality is important for various applications, which is determined by the aperture width and the beam divergence. For a high-power multimode laser, the output beam along the vertical direction (perpendicular to the layers of the heterostructure) is close to the ideal case of a diffraction-limited beam, while along the lateral direction (parallel to the layers), it strongly deviates from the ideal. That is why a widely used characteristic of the beam quality of a multimode laser diode is the lateral brightness $B_{lat}$ [6,7,8], which is defined as follows:

(2) Blat=PoutBPPlat

where $P_{out}$ is an output optical power and ${BPP}_{lat}$ is a lateral beam parameter product. The ${BPP}_{lat}$ parameter is determined by the presence of high-order modes and is quantified as follows:

(3) $${BPP}_{lat}=\frac{w_{lat}}{2}*\frac{\theta }{2}$$

where $w_{lat}$ is a lateral near field width and θ is a lateral beam divergence. The lateral near field width is determined by the stripe width, but at high pump current densities, it can exceed the nominal stripe width W due to current spreading. It is convenient to determine the lateral width and divergence using the intensity level of 1/e^2^ (13.5%) of the maximum, since this approach takes into account most of the power contained in the beam.

According to Equation (2), it can be seen that the brightness is determined, firstly, by the power, which is directly proportional to the stripe width according to Equation (1), and secondly, by the lateral beam parameter product ${BPP}_{lat}$, which, according to Equation (3), is also proportional to the stripe width. Thus, from these considerations (Equation (2)), it can be seen that, to a first approximation, a change in the stripe width does not affect the brightness. However, in practice, one can observe the brightness on the stripe width dependence, since the effect of the stripe width on the physical processes in the laser is more complex than that described by Equations (1)–(3).

The combination of various characteristics and parameters of the laser determines its application field. In the cases of fiber or solid-state laser pumping, as well as flash LIDARs [9] and laser illumination systems, the highest light power is required with moderate beam quality requirements, i.e., wide aperture lasers are required. For a number of applications (telecommunications, ranging and scanning systems, and energy transfer) that require high beam quality, the use of a wide-aperture laser is either impossible or requires beam masking with corresponding efficiency losses of the entire system. Thus, lasers with different aperture widths are demanded for different objectives, and choosing an inappropriate laser aperture can lead to inefficient use or insufficient performance of a particular system.

The present study is devoted to the creation of a set of 1550 nm multimode diode lasers with different stripe widths, fabricated on the basis of a single heterostructure. The main objective is to study the electro-optical characteristics of these lasers, in particular, the effect of the stripe width on the beam spatial parameters and brightness. The stripe width ranges from 20 to 150 μm, which is caused, on the one hand, by the implementation of a multimode operation, and, on the other hand, by the technological limitations of precision mounting of a wide-stripe chip on a heat sink.

## 2. Experimental Samples

The InGaAs/AlGaInAs/InP material system was used in a MOCVD-grown heterostructure to provide lasing at 1550 nm [1]. The active region was formed by two compressively strained (~1%) AlGaInAs quantum wells. Within such an approach to the active region design, the probability of Auger recombination processes and the interband absorption of charge carriers in the valence band decreases [10,11]. Layers with mechanical strain of opposite signs were used to compensate the strain in the active region. The active layer was located in the center of an ultra-narrow waveguide consisting of AlGaInAs-AlInAs graded layers. With such a composition, the process of thermal escape of charge carriers from the active region into the waveguide was suppressed, and with the ultra-narrow design, the heat dissipation from the active region improved and the accumulation of charge carriers in the waveguide was reduced, which was the power limitation’s main cause [10,12,13]. The waveguide was placed between the InP cladding layers. Special barrier layers made of the AlInAs wide-gap material were introduced in order to reduce the leakage of charge carriers from the waveguide into the cladding layers [14,15]. With such a design, there was a zero-order transverse mode, and the main fraction of the electromagnetic wave propagated in the InP cladding layers, in which the carrier density changed insignificantly. The cladding layers have a graded doping profile to provide efficient carrier injection and transport, minimal series resistance and low internal optical loss. A heavily doped InGaAsP layer was included in the central region of the upper p-emitter (at a depth of about 1.5 μm), which acts as a stop layer during the formation of the active emitter.

The next important step in the creation of a semiconductor laser is the post-processing operation of the active emitter. The active emitter is designed to suppress the lateral current spreading and to confine lateral modes. The mesa-stripe design, in which oblique grooves are etched through the heterostructure layers, is the simplest for creating a lateral waveguide. We use two types of mesa, “shallow” and “deep”, which differ in the depth of groove etching [13,16]. Each design has its advantages and disadvantages, so the choice of active emitter design depends on specific requirements.

There are both the complete elimination of the lateral current spreading and the ultra-low threshold current within the deep mesa design approach with grooves etched through the active region and the waveguide. In addition, for the deep oblique mesa design, there is no lasing quenching, which arises as a result of closed-loop modes lasing [17]. On the other hand, for the “deep mesa” design, the near field is observed to be inhomogeneous, which can lead to premature catastrophic optical mirror degradation, and the far field is observed to be non-ideal with a wide angular beam divergence. The “deep mesa” design can be used in research or applications where operation at high pump current levels is required.

We use the “shallow mesa” design in applications where beam quality is an important factor. The “shallow mesa” is etched in the upper p-cladding in order to avoid defects in the waveguide layer and active region, as well as side wall surface recombination. A laser of the “shallow mesa” design shows lower lateral divergence. However, it less effectively confines current spreading, and at high levels of current injection, this design may lead to closed-loop mode lasing. It is possible to control the closed-loop modes and to control the current spreading and the lateral divergence by controlling the etching depth. It is also possible to prevent the lasing quenching in a shallow mesa structure by means of various design and technological solutions presented in [18,19,20,21].

To address the objective of developing a set of semiconductor lasers suitable for various applications, a specific design for the active region was chosen, known as the “shallow mesa,” with stripe widths of 20, 40, 60, 100 and 150 μm. The mesa etching depth was calculated based on the requirements for low lateral field divergence and efficient current confinement. To facilitate precise and convenient mesa-groove etching, an InGaAsP stop layer was incorporated into the heterostructure design, specifically within the upper p-cladding region. Its position corresponded to the required etching depth. The stop layer, with its slower etching rate, effectively halted the etching process of the cladding at the intended location. The etching depth and stripe width were controlled using a scanning electron microscope (SEM) model “Camscan” 4-88-DV-100.

**Figure 1:**
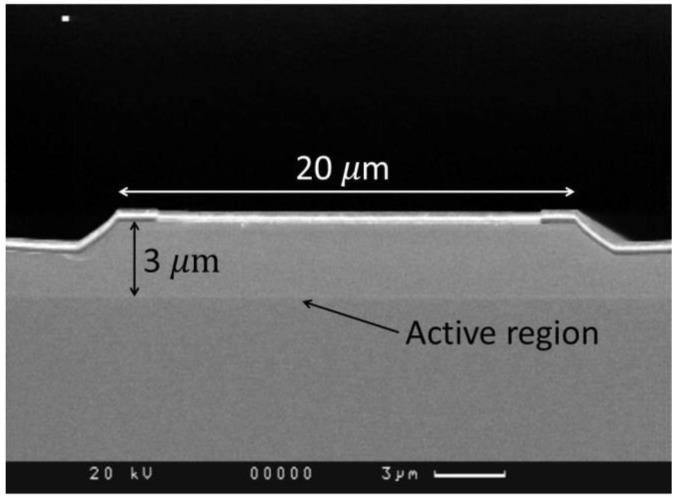
SEM image of the laser front facet with a stripe width of 20 µm.

After the mesa stripe formation, the passive (unpumped) regions of the heterostructure were coated with a dielectric material. An ohmic p-contact was then formed on the stripe. The n-type substrate was thinned down to 150 μm to reduce the laser series resistance, and an ohmic n-contact was formed on it. Figure 1 shows the SEM image of the front facet of the completed laser chip with a stripe width of 20 µm and mesa grooves with oblique walls.

Since the laser cavity parameters have an impact on the threshold current density, external differential efficiency and other output characteristics, special attention was given to selecting the optimal cavity length. For each stripe width, the standard characterization of lasers of various lengths with “as cleaved” mirrors (without coatings) was carried out: measurements of the light–current curves (L-I) and current–voltage curves (I-V) in quasi-continuous mode. Based on the obtained experimental data, the optimal cavity length for lasers with stripe widths ranging from 20 to 150 µm was found to be in the range of 2300–2700 µm, considering the best power characteristics. The optimal cavity length values coincide with the results obtained in [5,22]

Based on these results, the processed wafers that underwent all the stages of post-processing operation were cleaved into laser bars of optimal length (approximately 2500 µm). To allow emission through only front mirror, layers of Si and Si/SiO_2_ were deposited to form anti- (AR) and highly reflective (HR) coatings. Subsequently, the laser bars were cleaved into single dies and soldered onto copper heat sinks with the heterostructure layers down. Thus, using the “shallow mesa” technique, lasers were fabricated with stripe widths of 20, 40, 60, 100 and 150 µm; a cavity length close to 2500 µm; and facet reflectivities of 5% and 95%.

## 3. Measurements

For all fabricated lasers, L-I and I-V measurements, as well as measurements of the lateral and vertical far field beam divergences, the lasing spectra and the near fields were carried out in continuous-wave (CW) mode of laser operation at a heat sink temperature of 25 °C. A thermal stabilization system consisting of a Peltier element, a water-cooled radiator and a temperature sensor was used to maintain the laser temperature with an accuracy of 0.1 °C.

**Figure 2:**
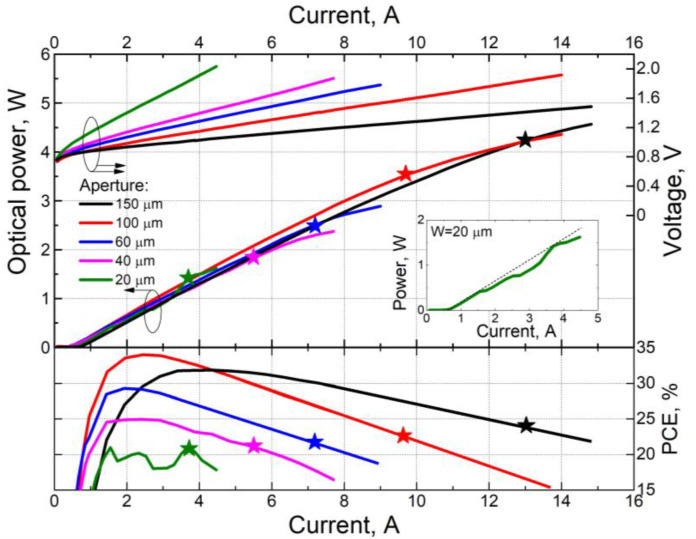
L-I, I-V and efficiency curves of lasers with AR and HR coatings; a cavity length of about 2500 µm; and a stripe width of 20, 40, 60, 100 and 150 µm.

Figure 2 shows the representative L-I and I-V curves for lasers with AR and HR coatings; a cavity length of about 2500 μm; and a stripe width of 20, 40, 60, 100, and 150 μm. The cutoff voltage, which was determined at the point of intersection of the tangent to the I-V curve on its linear part and the V-axis, was approximately 0.8 V for all samples. The series resistance was defined as the slope of the tangent to the I-V curve. As the stripe width increases, the series resistance decreases due to the larger contact area of the laser.

The L-I curves are smooth for all lasers, except for lasers with a stripe width of 20 µm. The optical power sharp bends or hops (“kinks”) are observed for the lasers with a stripe width of 20 µm (inset in Figure 2). The position of the kinks on the L-I curves is relatively stable (it does not change during repeated measurements), which means that they are related to the peculiarities of the operation of such lasers. For lasers with a stripe width of 20 µm, one can treat it as a low-mode lasing regime [6] and kinks in the L-I curve indicate switching between modes. The onset of kinks can be caused by various factors, such as the inhomogeneity of the active medium, the nonlinear optical properties of the material, saturation effects and others. The features of the operation of lasers with an aperture of 20 µm and the causes of the kinks onset will be considered in more detail in a separate future study.

At the initial segment of the I-V curve, with the same pump current values, the optical output power is similar for all samples, and the dependence on the stripe width is weak. Further, as the pump current is increased, the power dependence on the stripe width becomes evident. With an increase in stripe width, the area of the diode increases, and the active region overheating decreases. Due to the lower active region overheating, the deviation from the linearity of the I-V curve decreases, allowing for higher optical output power, as predicted by Equation (1).

Figure 2 shows the power-conversion efficiency (PCE) as a function of the pump current for the lasers under study. The laser efficiency is not directly related to the stripe width; it is related to the laser design through the external differential efficiency (the slope of the L-I curve), the threshold current density and the series resistance (the slope of the I-V curve). As the stripe width increases (from 20 to 100 µm), the maximum efficiency also increases due to the reduction in threshold current density and series resistance, reaching 34% for lasers with a 100 µm stripe width. However, further increasing the stripe width to 150 µm leads to a decrease in the maximum efficiency, primarily due to a decrease in external differential efficiency. Moreover, for a stripe width of 150 µm, the efficiency maximum shifts towards higher currents, and its dependence on the pump current (for currents exceeding 5 A) becomes less pronounced due to the low series resistance. Consequently, at powers above 1.5 W, lasers with a 150 µm aperture prove to be more efficient than others.

Based on the obtained results, for each sample, the operating current was determined, as a current at which the best balance between power and efficiency is achieved (on the figures, the operating current is indicated by “star” symbols). The operating current and characteristics of all lasers measured at operating current are presented in Table 1.

**Figure 3:**
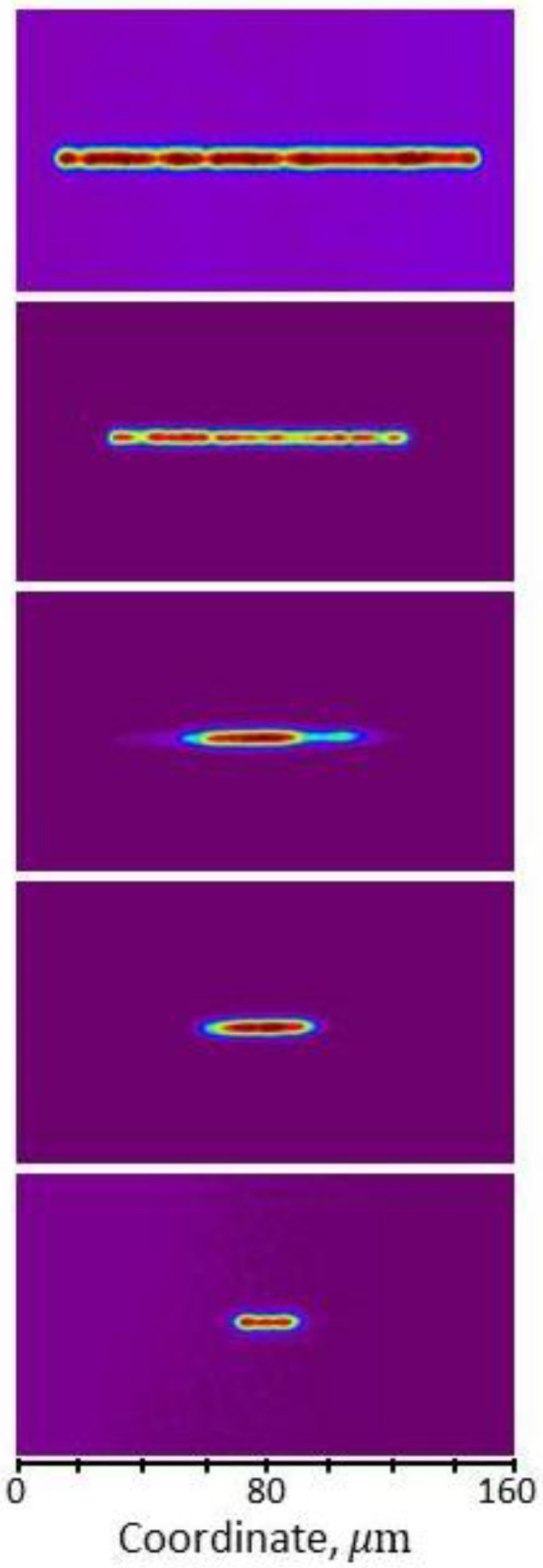
Near fields at the operating current for lasers with a stripe width of 20, 40, 60, 100 and 150 µm.

Subsequently, at the operating current, the near fields of lasers were obtained (Figure 3) using a lens system and a phosphor-coated silicon CCD high-resolution camera Ophir-Spiricon SP620U-1550 (Ophir Photonics, Jerusalem, Israel). The lateral dimensions of the near fields were measured, and the results are presented in Table 1. The somewhat larger spot sizes compared with the etched stripe width are associated with a minor current spreading in the “shallow mesa” design.

**Figure 4:**
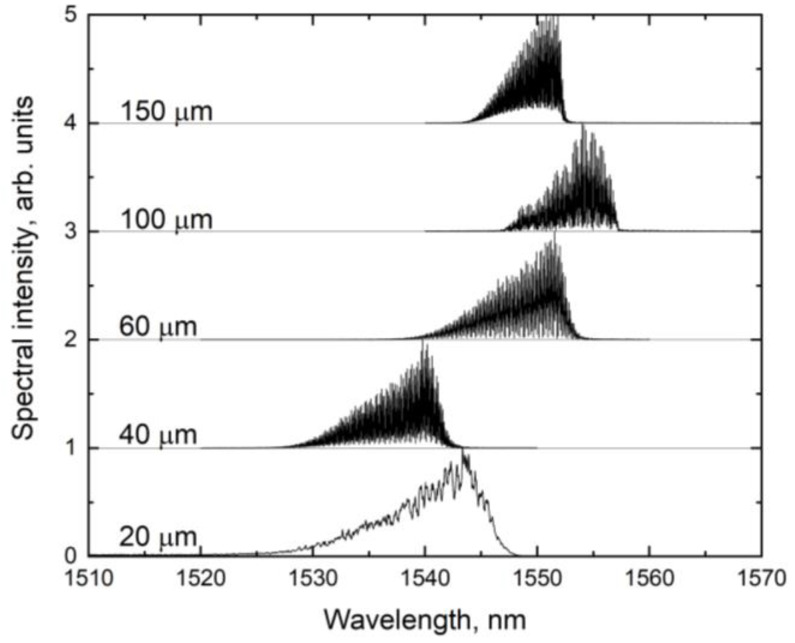
Laser spectra at operating current for lasers with stripe widths of 20, 40, 60, 100 and 150 µm.

The lasing spectra were measured in the CW mode at the operating current. The results are shown in Figure 4. The emission wavelength of lasers based on this heterostructure was 1550 ± 10 nm; the small wavelength spread can be explained by slightly different parameters of the samples. All spectra have a regular shape with a sharp long-wavelength edge and a smooth short-wavelength edge.

**Figure 5:**
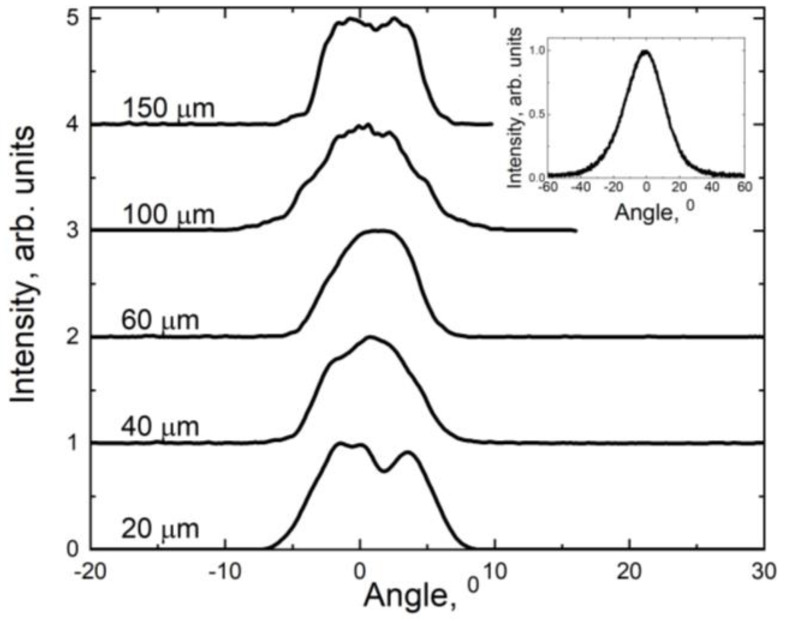
Lateral far fields at operating current for lasers with stripe widths of 20, 40, 60, 100 and 150 µm. A vertical far field is shown in the inset.

The lateral and vertical far fields were measured for the samples. All lasers are made from the same wafer, so they have the same vertical far-field intensity distribution, which corresponds to the electromagnetic wave field profile in the waveguide. All lasers operate in the fundamental mode in the vertical direction under all pump currents. The inset of Figure 5 shows a typical far field. The laser beam divergence was defined as full-width at half-maximum (FWHM). The vertical FWHM is about 26–30°. Figure 5 shows typical lateral far fields. The emission is multimode in the lateral direction. The lateral FWHM is in the range of 6–10°. The lateral beam divergences at the 1/e^2^ level are presented in Table 1.

Thus, all the main characteristics of lasers with different aperture widths were measured. With the data obtained, it is possible to estimate the beam quality dependence on the stripe width (Table 1). For the heterostructure design under study, as the stripe width increases, the ${BPP}_{lat}$ noticeably increases, which indicates an increase in the number of higher-order lateral modes that contribute to the power. However, it can be seen that ${BPP}_{lat}$ increases disproportionately to an increase in the stripe width (when the stripe width changes by a factor of 7.5, ${BPP}_{lat}$ changes by five times). This may be due to the fact that ${BPP}_{lat}$ is strongly affected by current-induced heating and the associated thermal lens formed inside the laser. When charge carriers are injected, the local temperature increases and temperature gradients resulting from heat diffusion cause modulation of the refractive index distribution and contribute to the formation of a lateral positive lens [23,24]. This thermal lens controls the lateral waveguide and influences the propagation of lateral optical modes.

As follows from Table 1, the lateral brightness $B_{lat}$ at the operating point of each laser slightly decreases with the stripe width increase. As mentioned above, in the first approximation, the brightness does not depend on the stripe width. However, in this case, a dependence is observed. This can be due both to disproportionate ${BPP}_{lat}$ dependence on the stripe width and to disproportionate optical power dependence on the stripe width. For example, with a stripe width increase, the profile of heat dissipation from the stripe changes noticeably, and it also becomes more difficult to create a high-quality thermal contact when soldering the die onto the heat sink, which in total leads to poor heat dissipation from the active region of wide-aperture lasers and, accordingly, to a power decrease compared with the prediction of Equation (1).

## 4. Conclusions

As a study result, an AlGaInAs/InP heterostructure with an ultra-narrow waveguide was designed and fabricated. Post-growth processing was carried out, resulting in a set of semiconductor lasers with different stripe widths. Measurements of the I-V and L-I curves, and spectral and spatial characteristics of the laser radiation were conducted. It was demonstrated that with an increase in the stripe width from 20 to 150 µm, the operating optical power increased from 1.4 W to 4.3 W. However, simultaneously, the lateral brightness decreased from 1.09 W/(mm*mrad) to 0.65 W/(mm*mrad). For lasers with a stripe width of 100 µm, a maximum efficiency of 34% was achieved, while the overall efficiency at the operating current for all samples was approximately 21–24%. The obtained values of the optical power and efficiency for the presented lasers corresponded to the world-class performance for lasers with emission wavelengths near 1550 nm [1,25].

From the presented set of lasers with different stripe widths, it is possible to select a laser with characteristics suitable for specific applications.

## Figures and Tables

**Table 1:** Key characteristics of the fabricated lasers at the operating current.

Stripe Width/Aperture	20 µm	40 µm	60 µm	100 µm	150 µm
Operating current, A	3.7	5.5	7.2	9.7	13
Power, W	1.42	1.88	2.5	3.5	4.3
WPE, %	21	21	21,5	22,5	24
Lateral near-field width, µm	24	43	66	103	154
Lateral far-field angle (1/e^2^), degrees	12.4	10.5	9.8	11.9	9.8
Wavelength, nm	1544	1540	1550	1554	1550
${BPP}_{lat}$, mm*mrad	1.3	1.97	2.82	5.35	6.61
$B_{lat}$, W/(mm*mrad)	1.09	0.95	0.88	0.65	0.65

## Data Availability

The data presented in this study are available from the corresponding author upon request.
